# The interplay of phloem-mobile signals in plant development and stress response

**DOI:** 10.1042/BSR20193329

**Published:** 2020-10-06

**Authors:** Amanda M. Koenig, Susanne Hoffmann-Benning

**Affiliations:** Department of Biochemistry and Molecular Biology, Michigan State University, East Lansing, MI 48824, U.S.A.

**Keywords:** lipids, Long-distance signaling, nucleic acids, phloem, plant development and stress response, proteins

## Abstract

Plants integrate a variety of biotic and abiotic factors for optimal growth in their given environment. While some of these responses are local, others occur distally. Hence, communication of signals perceived in one organ to a second, distal part of the plant and the coordinated developmental response require an intricate signaling system. To do so, plants developed a bipartite vascular system that mediates the uptake of water, minerals, and nutrients from the soil; transports high-energy compounds and building blocks; and traffics essential developmental and stress signals. One component of the plant vasculature is the phloem. The development of highly sensitive mass spectrometry and molecular methods in the last decades has enabled us to explore the full complexity of the phloem content. As a result, our view of the phloem has evolved from a simple transport path of photoassimilates to a major highway for pathogens, hormones and developmental signals. Understanding phloem transport is essential to comprehend the coordination of environmental inputs with plant development and, thus, ensure food security. This review discusses recent developments in its role in long-distance signaling and highlights the role of some of the signaling molecules. What emerges is an image of signaling paths that do not just involve single molecules but rather, quite frequently an interplay of several distinct molecular classes, many of which appear to be transported and acting in concert.

## Introduction

The vascular system is fundamental for systemic transport of energy-rich molecules, building blocks, and nutrients in the plant. It comprises the xylem for unidirectional translocation of water and minerals from roots to shoots and the phloem, whose predominant role is the transport of photoassimilates from source to sink tissues. The xylem pulls water and minerals up through xylem vessels, driven by the water potential gradient between the soil and the atmosphere surrounding the plant. The phloem consists of three main components: the companion cells (CCs), the sieve elements (SEs), and the parenchyma cells. The main conduit for phloem transport, the SEs, are enucleated cells separated by porous sieve plates, through which long-distance transport occurs by bulk flow according to Muench’s Pressure-Flow Hypothesis [1]. The Pressure-Flow Hypothesis attributes phloem transport to the difference in osmotic pressure between source tissues with high concentrations of sugars and sink tissues with low sugar concentration. While the SEs have few organelles and are thought not to perform transcription or translation, the neighboring CCs are fully functional cells that load and unload photoassimilates and other macromolecules into and out of the SEs. The loading of photoassimilates can follow several paths, namely, active apoplastic transport facilitated by Sugar Will Eventually be Exported Transporters (SWEETs) and Sucrose Transporters (SUTs); passive symplastic loading through plasmodesmata (PD) between mesophyll, CCs, and the SE; or polymer trapping dependent on the generation of a concentration gradient via the synthesis of raffinose and stachyose from sucrose [2–5]. Unloading occurs through and is controlled by specific PD [6,7]. Beyond sugars, the phloem is also a conduit for remobilization of carbon and nitrogen from source tissues to sink tissues, often in the form of amino acids [8,9]. These processes occur, for example, during leaf senescence but also as a consequence of *de novo* amino acid synthesis [9]. Amino acids are thought to be loaded into the phloem via the apoplastic mechanism, regulated by transporters like Usually Multiple Acids Move In and Out Transporters (UMAMITs) for export out of leaf cells and Amino Acid Permeases (AAPs) for import into the phloem. Whereas symplastic mechanisms seem to predominate during unloading, with amino acids passing through PD from the SEs into sink tissues [8]. As a result, the phloem is critical for the reallocation of carbon and nitrogen assimilates for the systemic distribution of energy and nutrients in the plant.

Another critical function of the phloem is long-distance signaling, which is necessary for the systemic coordination of plant development under normal conditions as well as during abiotic stress. It is also essential for the response to biotic factors, such as viruses, mutualistic and pathogenic microbiota, fungi, herbivores, and other pathogens such as nematodes and sucking/piercing insects. Our knowledge of phloem anatomy, development, and function was thoroughly reviewed in 2013 [10]. Here we will focus specifically on the review and update of various phloem mobile macromolecules and their roles as systemic signals.

### Mobile macromolecules and the regulation of their transport

In addition to photoassimilates, other macromolecules, including nucleic acids, peptides, proteins, hormones, and lipids have been identified in the phloem [11,12]. Their presence elevates the function of the phloem from simple energy transport to an integral signaling conduit.

#### Nucleic acids

Nucleic acids, more specifically RNA species, are among some of the most well-studied phloem-mobile signaling molecules. For a more detailed review of RNA trafficking, see recent publications by Ham and Lucas [13], Lin and Chen [14], and Kehr and Kragler [15]. Representatives of all types of RNA, such as mRNA, microRNA (miRNA), small interfering RNA (siRNA), and other non-coding RNA (ncRNA) have been identified in the phloem of many plant species, including *Arabidopsis*, rice, barley, pumpkin, watermelon, cucumber, and several others [16–24]. While the existence of mRNA and ncRNA in the vasculature system hints at their mobility and possible role in plant development, identification alone does not conclusively prove movement or physiological function. Using predominantly grafting approaches, numerous studies have provided evidence for phloem-mobile mRNA [25,26] and ncRNA [27–30]. Furthermore, data suggest environmental conditions may affect transcript mobility independent of changes in gene expression, implicating mobile transcripts as possible stress response signals [26,31].

While some evidence suggest that mRNA mobility can be ascribed to transcript abundance and stability [32], other studies have identified selective mechanisms, including specific sequence motifs, that determine systemic transport of nucleic acids [33–36]. The untranslated regions of *StBEL5* appear to influence transcript stability for transport and impact translation and delivery to distal tissues [33]. Similarly, a *cis*-acting element within the 102 nucleotides at the 5′ end of *FLOWERING LOCUS T (FT)* is critical for the systemic transport of *Arabidopsis FT* RNA [37]. RNA-binding proteins play an important role in the translocation of some RNA species in the phloem; they contain motifs, like CU-rich polypyrimidine-binding regions, which can facilitate RNA binding and transport [34,35,38]. Additionally, other sequence motifs and secondary structures, such as tRNA-like structures, may also promote transcript mobility in the phloem [39]. Degradation mechanisms at the destination organs have also been suggested as the driving force for transcript signal selection, a hypothesis that further expands on the role of transcript stability for long-distance signaling [40].

Phloem-mobile RNA species show directionality, target specificity, and stress responsiveness. Further characterizations of selected phloem-localized RNA species have linked RNA to particular stresses and developmental signals.

#### Hormones and lipids

The phloem has been implicated in the systemic translocation of phytohormones, including auxins, gibberellins (GAs), cytokinins (CKs), jasmonic acid (JA), and abscisic acid (ABA) [41–44]. As a conduit for photoassimilates, the phloem is an aqueous, hydrophilic environment, and yet hydrophobic compounds including fatty acids, hormones, and lipids have been identified in the phloem [12,45,46]. While some of these hormones and other molecules may act as independent mobile signals or translocate as conjugates, lipophilic compounds may interact with proteins to facilitate their transport and signaling activity [45,47,48]. Protein-associated long-distance lipid transport is a widely accepted mechanism for signaling in animal systems [49–52]. Lipid interactions are necessary for the activation and function of transcription factors (TFs), interaction with receptors, and other signaling components, but have been understudied in plants [53–58]. However, several examples of lipid interaction with TFs have recently been published in plants as well: phospholipid–TF complexes play roles in flowering [59,60], the circadian clock [61], nuclear localization [62], and lipid metabolism [63]. Hence, some roles for protein–lipid complexes in systemic signaling in plants echo those in animal systems.

#### Proteins and peptides

Several proteomics studies have identified hundreds up to thousands of proteins in the phloem, despite little evidence for translation in the sieve tube, which suggests that peptides and proteins are loaded for transport as possible systemic signals [23,64–68].

It has been debated whether these non-sugar macromolecules diffuse into the phloem by accident [69–71] or enter through targeted transport [72,73]. At least in some cases these macromolecules have been shown to be carefully regulated, intentionally transported, and essential for successful plant development. For example, when proteins from pumpkin phloem exudate were introduced into the rice sieve tube, some proteins were transported shootward in the direction of bulk flow, whereas some moved rootward. This suggests that movement was destination-driven with a possible regulatory role for interacting proteins [74]. The PD likely play a crucial regulatory role in the loading and unloading of macromolecules into and out of the phloem stream, as they are known to mediate transport between cells [75]. Comparative proteomics has identified fluctuations in phloem protein composition under various environmental conditions, providing further evidence for controlled trafficking [66,76].

One of the specifically regulated and important regulatory proteins, FT (20 kDa), for example, is phloem-mobile and exhibits essential, tissue-specific function [77–79]. FT export into SEs for long-distance translocation is partly dependent on FT INTERACTING PROTEIN 1 (FTIP1), suggesting a mechanism for selective protein transport into the phloem [80]. Additionally, an MCTP-SNARE (multiple C2 domain and transmembrane protein-soluble *N*-ethylmaleimide-sensitive factor protein attachment protein receptor) complex composed of SYNTAXIN OF PLANTS 121 (SYP121) and QUIRKY (QKY) acts in a mechanism parallel to the FTIP1-mediated loading to transport FT into the SE [81]. Further regulation during phloem unloading at distal tissues or binding to spatially specific receptors, may contribute to systemic transport and specificity for delivery of physiologically relevant signals [82]. Taken together, these findings suggest tightly regulated and highly selective processes for the systemic trafficking of mobile signals like FT and other signaling proteins outlined in Table 1. While non-specific diffusion may account for some proteins in the phloem, it is evident that multiple mobile proteins, critical for development and stress response, are deliberately transported. These findings, along with convincing case studies of mobile proteins with physiological function, to be discussed throughout this review, demonstrate the controlled, purposeful translocation of systemic protein signals in the phloem

**Figure 1:**
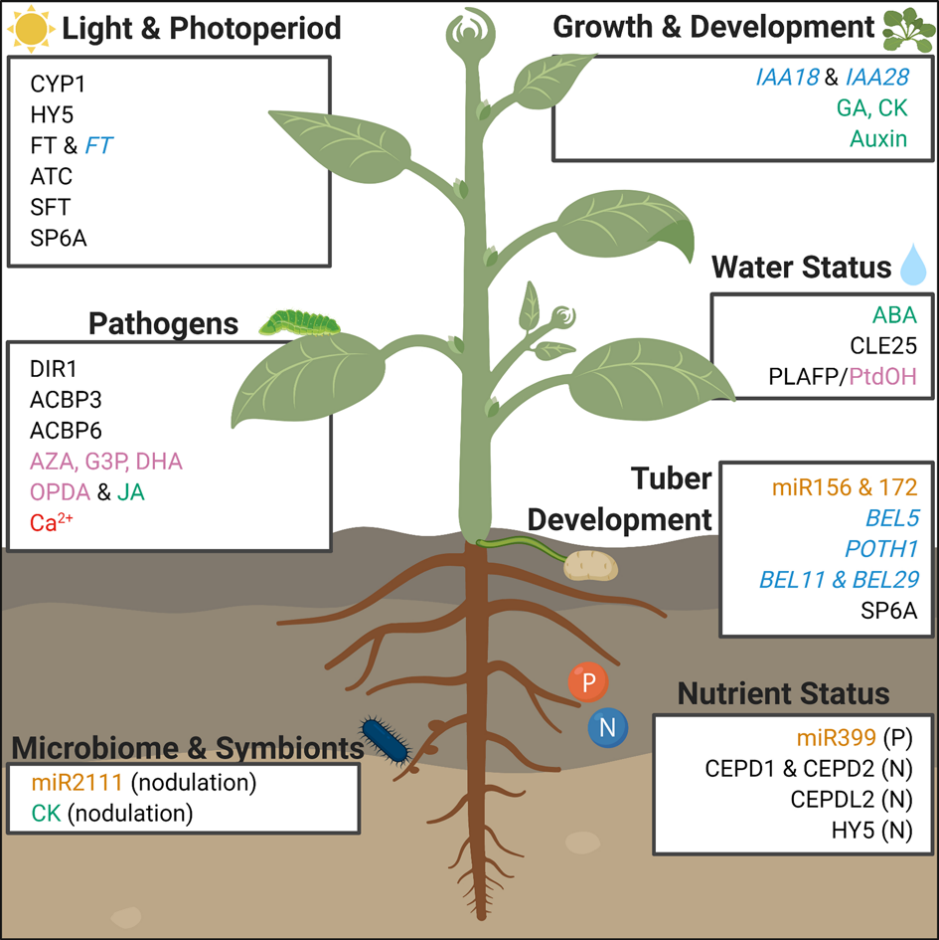
Overview of long-distance signaling in the phloem Mobile signals involved in plant development and stress response are listed: proteins (all-caps; black), lipophilic compounds (pink), ions (red), miRNA (orange), mRNA (italicized; light blue), hormones (green). Abbreviations, plants species, and references for all compounds are detailed in Table 1.

Table 1 lists all phloem-associated systemic signals by substance class with references and the plant species they were observed in. An overview of these phloem-associated systemic signals and their roles in plant development is provided in Figure 1.

**Table 1:** Phloem-localized systemic signals

Mobile signal	Abbreviation	Species class	Developmental process	Plant species	References
*Transition from vegetative to generative growth*					
FLOWERING LOCUS T	FT	Protein	Flowering	*Arabidopsis thaliana, Cucurbita moschata, Curcurbita maxima, Oryza sativa, Solanum lycopersicum, Zea mays, Glycine max, Nicotiana tabacum, Gossypium hirsutum, Solanum tuberosum*	[77,78,82,83–94,95]
		Nucleic acid (mRNA)			[37,96,97]
CENTRORADIALIS	ATC	Protein	Flowering	*Arabidopsis thaliana*	[98]
Gibberellins	GA	Hormone	Flowering	*Arabidopsis thaliana, Lolium temulentum*	[41,42,99,100,99–110]
Cytokinins	CK	Hormone	Flowering	*Arabidopsis thaliana, Elaeis guineensis*	[41–44,100]
SINGLE FLOWER TRUSS	SFT	Protein	Flowering, leaf maturation, termination, and abscission	*Solanum lycopersicum*	[87,88,111]
*Tuber development*					
*BEL1-RELATED HOMEOTIC PROTEIN 5*	*BEL5*	Nucleic acid (mRNA)	Tuber development (promotion)	*Solanum tuberosum*	[112]
*BEL1-RELATED HOMEOTIC PROTEIN 11*	*BEL11*	Nucleic acid (mRNA)	Tuber development (inhibition)	*Solanum tuberosum*	[113]
*BEL1-RELATED HOMEOTIC PROTEIN 29*	*BEL29*	Nucleic acid (mRNA)	Tuber development (inhibition)	*Solanum tuberosum*	[113]
MicroRNA 156	miR156	Nucleic acid (miRNA)	Tuber development (promotion)	*Solanum tuberosum*	[114]
MicroRNA 172	miR172	Nucleic acid (miRNA)	Tuber development (promotion)	*Solanum tuberosum*	[115]
*POTATO HOMEOBOX 1*	*POTH1*	Nucleic acid (mRNA)	Tuber development (promotion)	*Solanum tuberosum*	[116]
SELF-PRUNING 6A	SP6A	Protein	Tuber development/photoperiod (promotion)	*Solanum tuberosum*	[95]
*Nutrient status*					
MicroRNA 399	miR399	Nucleic acid (miRNA)	Phosphate starvation	*Arabidopsis thaliana, Brassica napus, Cucurbita maxima*	[117,118]
C-TERMINALLY ENCODED PEPTIDE (CEP) DOWNSTREAM 1	CEPD1	Protein	Nitrogen acquisition	*Arabidopsis thaliana*	[119]
CEP DOWNSTREAM 2	CEPD2	Protein	Nitrogen acquisition	*Arabidopsis thaliana*	[119]
CEPD-LIKE 2	CEPDL2	Protein	Nitrogen acquisition	*Arabidopsis thaliana*	[120]
ELONGATED HYPOCOTYL 5	HY5	Protein	Nitrogen acquisition and carbon fixation	*Arabidopsis thaliana*	[121]
*Root development*					
CYCLOPHILIN 1	CYP1	protein	Photosynthetic status/light intensity, root morphology/nutrient acquisition	*Solanum lycopersicum*	[122,123]
*INDOLE-3-ACETIC ACID 18*	*IAA18*	Ncleic acid (mRNA)	Auxin signaling, lateral root formation	*Arabidopsis thaliana*	[124]
*INDOLE-3-ACETIC ACID 28*	*IAA28*	Nucleic acid (mRNA)	Auxin signaling, lateral root formation	*Arabidopsis thaliana*	[124]
Auxin	*-*	Hormone	Gravitropism/halotropism	*Arabidopsis thaliana*	[41,125,126]
MicroRNA 2111	miR2111	nucleic acid (miRNA)	Nodulation	*Lotus japonicus*	[127]
Cytokinins	CK	Hormone	Nodulation	*Arabidopsis thaliana, Lotus japonicus*	[41–44,128]
*Biotic stress*					
DEFECTIVE IN INDUCED RESISTANCE 1	DIR1	Protein	Biotic stress, pathogen defense	*Arabidopsis thaliana, Cucumis sativus*	[79,129]
Dehydroabietinal	DHA	Lipid	Biotic stress, pathogen defense	*Arabidopsis thaliana*	[12,130,131,132]
Azelaic acid	AZA	Lipid	Biotic stress, pathogen defense	*Arabidopsis thaliana*	[12,133,129,131,134,132]
glycerol-3-phosphate	G3P	Lipid	Biotic stress, pathogen defense	*Arabidopsis thaliana*	[12,135,133,131,132]
ACYL-COA BINDING PROTEIN 3	ACBP3	protein	Biotic stress, pathogen defense	*Arabidopsis thaliana*	[136,137]
ACYL-COA BINDING PROTEIN 6	ACBP6	protein	Biotic stress, pathogen defense	*Arabidopsis thaliana, Cucurbita maxima, Oryza sativa*	[138,136,137]
12-oxo-phytodienoic acid	OPDA	Lipid	Wounding, pathogen defense	*Arabidopsis thaliana*	[12,139,140,132]
Jasmonic acid	JA	Hormone	Wounding, pathogen defense	*Arabidopsis thaliana, Nicotiana attenuata, Solanum lycopersicum*	[139–144]
Calcium	Ca^2+^	Ion/electrical signal	Wounding, pathogen defense	*Arabidopsis thaliana*	[145]
*Abiotic stress*					
Abscisic acid	ABA	Hormone	Water status	*Glycine max, Xanthium strumarium, Ricinus communis, Citrus sinensis x Poncirus trifoliata, Solanum lycopersicum, Arabidopsis thaliana*	[146–152]
CLAVATA3/EMBRYO-SURROUNDING REGION-RELATED 25	CLE25	Protein	Water status	*Arabidopsis thaliana*	[153]
PHLOEM LIPID ASSOCIATED FAMILY PROTEIN	PLAFP	Protein	Water status	*Arabidopsis thaliana, Brassica oleracea*	[12,132,154,155]
Phosphatidic acid	PtdOH	Lipid	Water status	*Arabidopsis thaliana*	[12,132,154,155]

Species and references highlight studies in which the relevant signal is phloem-mobile and functions in the associated developmental process.

The variation among phloem-localized macromolecules begs the question: how does this diversity of compounds—nucleic acids, proteins, hormones, lipids, ions, and their complexes—function in plant systems? What physiological demands dictate which molecular species is required?

Phloem-mobile macromolecules act in a variety of processes to coordinate plant development under both normal and challenging conditions. Long-distance signals influence aspects of plant morphology and physiology by participating in diverse genetic, biochemical, and even epigenetic mechanisms [101]. In the following sections, systemic signals in the phloem and their impact on the genetic and biochemical regulation of plant development are discussed. What will become obvious is that, often, not a single macromolecule but a combination of different signal classes can trigger the developmental response.

## Transition to flowering: vegetative to generative growth

Major developmental shifts require the careful integration of external and internal cues. Long-distance coordination and systemic signaling have been linked to changes in leaf phenotypes including pinnation and lobe patterns, trichome development, GA-related leaf development, and palisade tissue shape to optimize light absorption [99, 100, 102–105]. Control of leaf morphology is among the first examples of phloem-mobile mRNAs influencing plant physiology. These foundational studies pointed to the ability of long-distance RNA transport to play a functional role in plant development.

One of the most important and dramatic developmental changes in plants is the transition from vegetative to generative growth. This transition is controlled by the photoperiod, which is sensed in the leaves. A mobile signal, often referred to as florigen, is required to transmit information about photoperiod from the leaves to the shoot apical meristem (SAM) where flower development can begin. Depending on the plant species, phytohormones like GA, CKs, and ethylene participate in the regulation of flowering, either independently, as phloem-mobile signals themselves, or in FT/CONSTANS (CO)-dependent mechanisms [106–110, 156]. In many plant species, the key florigenic signal is the protein FT. In *Arabidopsis*, long day conditions and circadian clock components promote the transcription of *CONSTANS* in the leaf vasculature, which then interacts with the *FT* promoter to initiate its expression [157]. Several studies have conclusively shown that FT protein or its homolog in other plants is mobile and is central to the transition to reproductive growth [77,78,83–94]. Upon arrival at the SAM, it interacts with the bZIP transcription factor FD to initiate expression of genes necessary for floral development [158]. Interestingly, specific Phosphatidylcholine (PtdCho) molecular species exhibit diurnal oscillations in the SAM; FT preferentially binds the species that predominates during the day. [59]. A recent structural characterization of *Arabidopsis* FT suggests PtdCho as a mediating ligand to facilitate FT/FD/14-3-3 protein interaction to form the Florigen Activation Complex (FAC) and its binding to DNA to promote flowering [60]. Conversely, *Arabidopsis* CENTRORADIALIS (ATC) may act to counterbalance FT activity for floral initiation. Like *FT, ATC* is expressed in vascular tissue and ATC is graft-transmissible, indicating long-distance mobility. ATC may compete with FT to interact with the FD, which then inhibits flowering [98].

**Figure 2:**
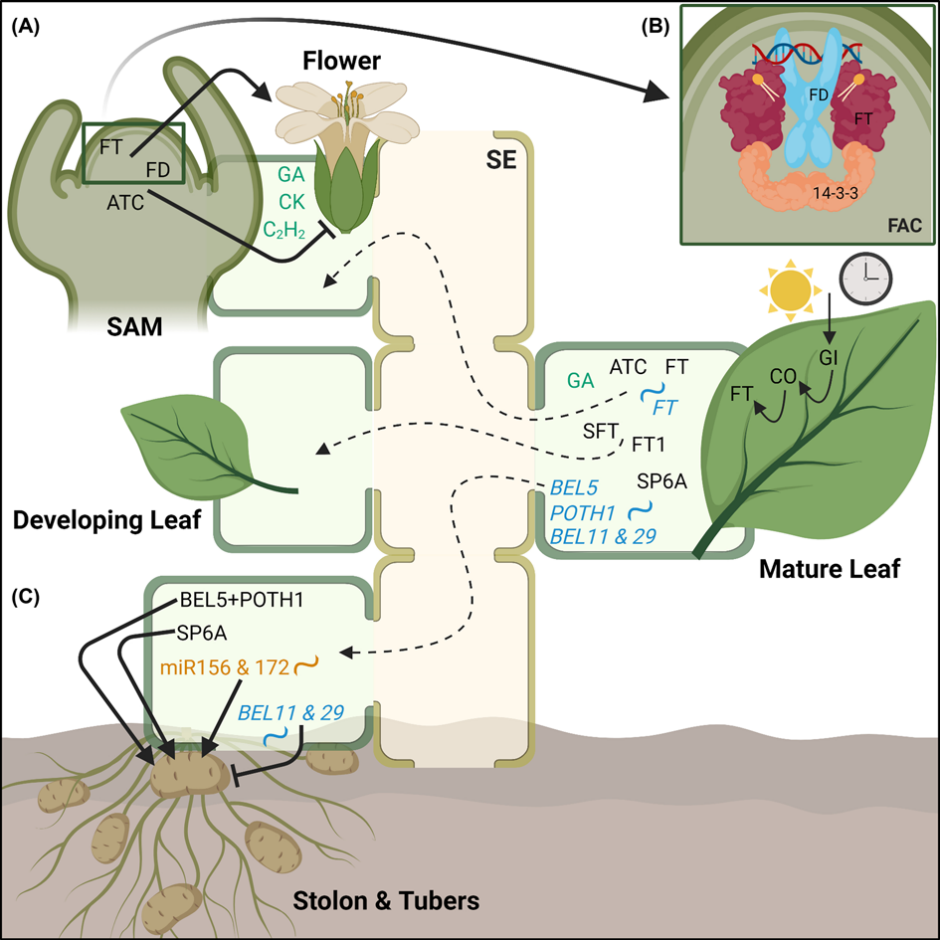
Phloem-mobile signals in shoot development (**A**) Transition to flowering: FT and its homologs are expressed in the leaves in a photoperiod-controlled mechanism through GIGANTEA (GI), other circadian clock components and CO. FT and FT-like proteins, as well as FT mRNA transcripts, are transported in the phloem to the SAM to promote the transition from vegetative to reproductive growth. FT interacts with FD to promote flowering, whereas ATC, a second mobile flowering regulator, competes with FT to inhibit flowering. (**B**) The FAC comprises FT, 14-3-3 protein, and the TF FD. Complex formation and binding to DNA is mediated by interaction with PtdCho, as shown by the cartoon of the proposed structure from Nakamura et al. [60]. Phytohormones GAs, CKs, and ethylene (C_2_H_2_) also play a role in flowering in some plant species. Other FT-like proteins, such as SFT (tomato) and FT1 (cotton), not only act in flowering but also in leaf maturation, termination, and abscission signaling. (**C**) Tuber formation: BEL5, 11, and 29 and POTH1 mRNA, as well as SP6A protein and miR156 and 172, move in the SE of the phloem to stolon tissue, where they regulate tuber development. A BEL5–POTH1 complex, SP6A, miR156 and miR172 promote tuber formation, whereas BEL11 and BEL29 suppress it. Abbreviations, plants species and references for all compounds are detailed in Table 1.

Despite extensive evidence for FT protein, more recent research also suggested movement of *FT* mRNA. Non-translatable *FT* RNA is independently phloem-mobile, facilitates the movement of other RNAs, and can overcome SAM viral exclusion mechanisms [37,96,97]. The regulatory mechanisms for the transition from vegetative to reproductive growth are summarized in Figure 2.

FT is pivotal for the transition from vegetative to generative growth. However, its role is not simply confined to the induction of flowering. FT and its homologs control additional seasonal and developmental responses (Figure 2). The mobile florigen hormone in tomato, SINGLE FLOWER TRUSS (SFT), affects leaf maturation, termination, and abscission [87,88,111]. Overexpression of the cotton FT-like gene *GhFT1* promotes lateral shoot outgrowth and early flower abscission [93]. These studies, along with other examples, highlight FT and FT-like proteins as crucial not only in flowering but also in the overall balance between vegetative and reproductive growth and development [159].

## Development of underground organs

### Tuber development

Plants develop several bulbous underground storage organs. Some are derived from roots; others however, despite their location, are derived from specialized shoots. Tuber formation in potato is coordinated, in part, by day length-associated signals in above ground tissue. Several phloem-mobile transcripts, including multiple *StBELs* and *POTATO HOMEOBOX 1* (*POTH1*) mRNAs as well as miR156 and 172, work in concert to regulate tuber formation in potato [112–116]. Facilitated by short-day induced RNA-binding proteins at its 3′ untranslated region, *StBEL5* transcripts are transported from leaves to stolon, where the StBEL5–POTH1 complex induces gibberellic acid-, CK-, and auxin-related genes for tuber formation [33,112,160–163]. Phylogenetically related transcripts *StBEL11* and *StBEL29* mirror *StBEL5* in phloem mobility and accumulation during short days; however *StBEL11* and *StBEL29* seem to act antagonistically to *StBEL5* and reduce tuber yield [113]. These three transcripts may work in conjunction to balance cell growth during the transition from stolon to tuber in potatoes and coordinate tuber formation according to photoperiod by translocating in the phloem from leaves to stolon. An FT ortholog in potato, SELF-PRUNING 6A (StSP6A), is another promising phloem-mobile candidate for photoperiod-regulated systemic control of tuberization and may be a target of *StBEL5* [95,164]. Mechanisms for tuber initiation and development, including these phloem-mobile players, are further evaluated and reviewed in [165–167]. Again, the interplay between multiple mobile species, mRNA, miRNA and proteins, defines converging paths and efficiency of long-distance signaling.

### Root development and nutrient deficiency

Root development is regulated by the availability of nutrients from the soil as well as the abundance of energy carriers and building blocks generated above ground. Similarly, nutrient availability affects shoot development as well. Hence, plants tightly regulate nutrient sensing, acquisition, and allocation to maintain their health and productivity. Phloem transport plays an important role in the systemic signaling of nutrient levels. For example, iron content in phloem is determined, in part, by the loading activity of OLIGOPEPTIDE TRANSPORTER 3 (OPT3) and iron itself influences the expression of Fe uptake genes like *IRON-REGULATED TRANSPORTER 1* (*IRT1*) and *FERRIC REDUCTION OXIDASE 2* (*FRO2*) in the roots, which then modulates Fe distribution between source and sink tissues along with other, as of yet, unidentified shoot-borne signals [168–170]. Furthermore, limited sulfur, copper, iron, and phosphorus availability affects proteins, miRNA and other small RNA species in the phloem [171–173]. As such, mobile RNAs and peptides are involved in communicating nutrition status and coordinating mineral allocation systemically.

**Figure 3:**
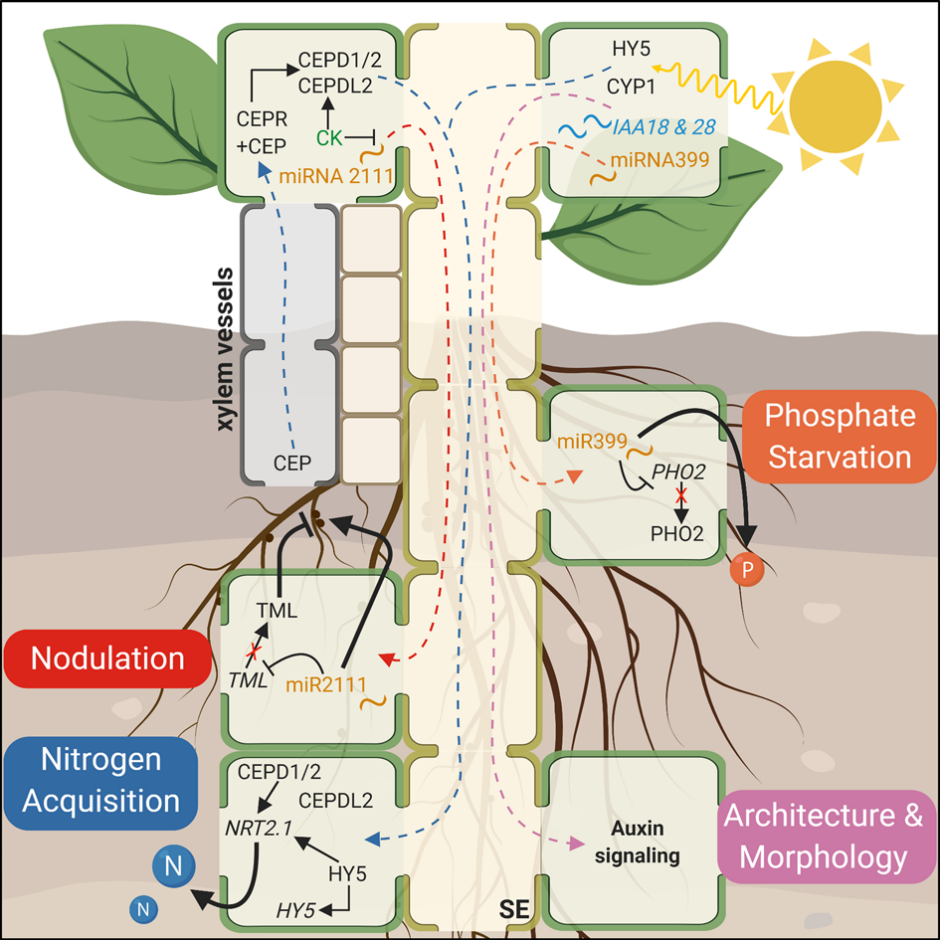
Phloem-mobile signals in root development Nodulation (red arrows): In unmodulated roots, shoot-derived miR2111 translocates to the roots, where it inhibits TML, thereby promoting susceptibility to nodulation. Following sufficient nodulation, a root-derived CK-dependent signal represses miR2111 production, which restricts further nodule formation. Nitrogen acquisition (blue arrows): Under nitrogen-starved conditions, CEP translocates in the xylem from roots to shoots, where it is sensed by its receptor, CEPR. The CEP–CEPR module promotes the production of CEPD1 and 2 proteins, and, together with CK, the expression of CEPDL2. These proteins are transported in the phloem to the roots to regulate the expression of the nitrogen transporters NRT2.1, thus influencing nitrogen uptake and transport. HY5 integrates carbon fixation and photosynthetic status with nutrient acquisition by translocating from leaves to roots, where it auto-activates its expression and promotes NRT2.1-mediated nitrogen uptake. During phosphate starvation (orange arrows), miR399 is expressed in the leaves and translocates in the SE to the roots, where it suppresses expression of PHO2, allowing an increase in phosphate uptake and loading. Architecture and root morphology (pink arrows): CYP1 and IAA 18 and 28 transcripts translocate from shoot to root, where they coordinate light and photosynthetic status in the leaves with root development and nutrient acquisition in the roots. Abbreviations, plants species and references for all compounds are detailed in Table 1.

Under **phosphate starvation**, plants employ several mechanisms to maintain homeostasis, including lipid remodeling as well as reallocation of excess phosphate from the roots to developing tissues [174,175]. Under Pi-deplete conditions in shoots expression of miRNA 399 (miR399) increases. The miR399 then translocates in the phloem from shoot to root where it suppresses *PHOSPHATE 2* (*PHO2*) expression, via the gene silencing mechanism. The absence of PHO2, an E2 ubiquitin-conjugating enzyme that represses phosphate uptake, allows for an increase in Pi loading into the xylem for transport to shoots (Figure 3, orange) [117, 118, 176–179]. In addition, under phosphate limited conditions, many mRNA transcripts, including hormone receptors, TFs, and P_i_ signaling genes like *PHO2*, as well as lncRNA transcripts like the target mimic of miRNA399 *INDUCED BY PHOSPHATE STARVATION 1* (*IPS1*), are differentially expressed in source phloem and subsequently transported to sinks—developing leaves, the shoot apex, and root tips—in a tissue-specific manner [31,34,40]. While the exact mechanism and function of these mobile transcripts is still unknown, it is clear that a variety of RNA species are involved in the systemic early response to phosphate deficiency by coordinating nutrient perception between roots and shoots.

Roots sense **nitrogen availability** in the soil and adjust their architecture to improve nutrient uptake. Systemic N-demand signaling involves a xylem-mobile peptide called C-TERMINALLY ENCODED PEPTIDE (CEP), which signals nitrogen starvation from the roots to the shoots [180,181]. CEP originates in the roots, accumulates in the vascular tissue in the leaf, and likely diffuses from the xylem into the phloem where it is sensed by CEP RECEPTOR (CEPR) protein. Downstream of the CEP-CEPR module, CEP DOWNSTREAM 1 and 2 (CEPD1 and CEPD2) are loaded into the phloem in the shoot and translocate to the roots. Once there, they increase the expression of NITRATE TRANSPORTER 2.1 (*NRT2.1*), but only if nitrate is available in the surrounding soil [119]. A third peptide, CEPD-LIKE 2 (CEPDL2) is expressed in the leaf vasculature in response to low nitrogen. In this case, both CKs and CEP appear to regulate gene expression. CEPDL2 then translocates to the root, where it affects both nitrate-uptake and transport through the regulation of the expression of several high-affinity nitrate transporters [120]. CEPD1/2 and CEPDL2 appear to be part of a dual N-sensing and response system, with CEPD1/2 responding to the root N status and CEPDL2 responding to the shoot status (Figure 3, blue). This root-to-shoot-to-root circuit utilizes both xylem and phloem to systemically coordinate nitrogen status across the whole plant.

Nitrogen acquisition in the roots is also informed by light cues and carbon fixation in the shoots. Multiple phloem mobile signals contribute to the integration of shoot-localized photosynthetic processes and nutrient uptake in the roots. The bZIP transcription factor ELONGATED HYPOCOTYL 5 (HY5), which regulates carbon assimilation in the shoots, translocates to the roots where it auto-activates its own expression [121]. The subsequent increase in HY5 TFs in the roots promotes *NRT2.1* expression and potentiates NRT2.1-facilitated transport of nitrate from the soil [121]. Shoot-derived CYCLOPHILIN 1 (CYP1) in tomatoes integrates information about light intensity and photosynthetic status in the shoots with morphology and nutrient acquisition in the roots by influencing auxin response (Figure 3, pink) [122,123]. In addition, auxin directly controls root architecture as well. The transcripts of *INDOLE-3-ACETIC ACID 18* (*IAA18*) and *IAA28*, which are expressed in leaf vasculature, were identified in *Arabidopsis* phloem and affect lateral root formation [124]. As has become clear in previous paragraphs, many developmental processes are not regulated by a single long-distance signal but rather an interplay of complementary signals, consisting of proteins, hormones, and nucleic acids.

### Nodulation

Plants are unable to fix atmospheric nitrogen on their own, so some species, namely legumes, form specialized root organs called nodules that function to facilitate mutualistic interactions with nitrogen-fixing soil bacteria. Root nodulation and symbiosis with rhizobia is controlled, in part, by systemic signaling, including a phloem-mobile miRNA species, microRNA 2111 (miR2111), that regulates susceptibility (Figure 3, red). *TOO MUCH LOVE* (*TML*), the target of miR2111, restricts nodule formation [127]. However, in the absence of rhizobia, miR2111 is expressed in leaf CCs and translocates to the roots, where mature miR2111 post-transcriptionally regulates *TML*, which down-regulates genes that affect nodulation factor perception and thus inhibits repression of nodulation [182]. Following exposure to rhizobia, a CK-dependent signal from roots inhibits miR2111 production in the shoot, which allows TML to limit nodule formation [127,128]. This long-distance circuit balances infection and nodulation for successful symbiosis.

## Biotics stress and systemic acquired resistance

**Figure 4:**
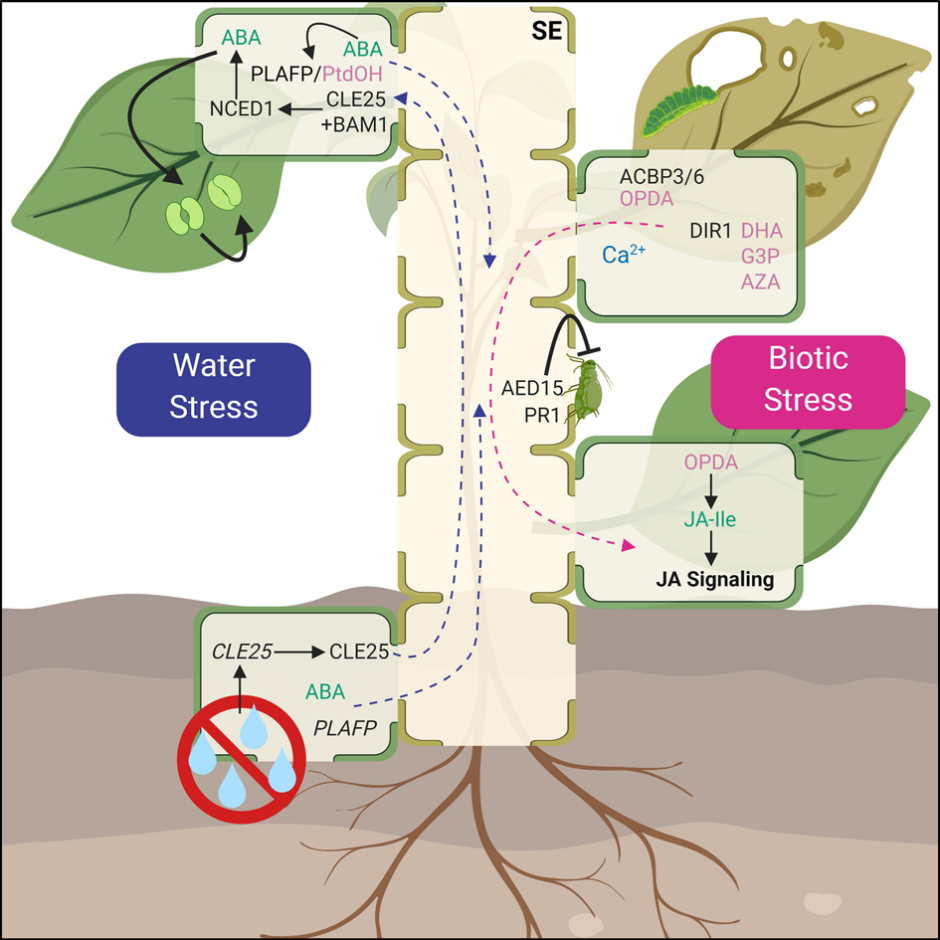
Phloem-mobile signals during water stress and biotic stress Water stress (blue arrows) signals, derived in both the roots and the shoots, are shown on the left. When water limitation is sensed in the roots, CLE25 translocates in the SE to leaf tissues and is perceived by BARELY ANY MERISTEM 1 (BAM1). The CLE25-BAM1 module promotes the expression NINE-CIS-EPOXYCAROTENOID DIOXYGENASE 3 (NCED3), which promotes synthesis of the phytohormone, ABA. ABA signaling regulates the closing of stomata (light green) in response to water stress. PLAFP and phosphatidic acid (PtdOH) are produced in response to ABA and may play a role in systemic drought response. Both ABA and PLAFP could be root- and/or shoot-derived. Biotic stress (magenta arrows; right half): AED15 and PR1 act directly as anti-herbivory or antimicrobial agents in phloem sap. Several proteins and lipophilic compounds, as well as electrical signals like Ca^2+^, are produced in wounded tissues and act in systemic signaling for resistance in distal tissues. ACBP 3 and 6 affect the fatty acid composition in the phloem, including oxylipins. The phloem-mobile, defense-response protein DIR1 may facilitate the transport of SAR signals like DHA into and through the phloem. Mobile signals such as DHA, AZA, or G3P derivatives move to distal tissues, where they can trigger defense response signals like JA signaling, involving the bioactive JA conjugate JA–Isoleucine (JA–Ile), and induce resistance systemically. Abbreviations, plants species, and references for all compounds are detailed in Table 1.

Pathogen infection triggers both a local and a systemic response. The latter often involves Systemic Acquired Resistance (SAR), a mechanism triggered by immune responses to local pathogen infection, which then allows distal tissues in plants to become resistant. While researchers have yet to unambiguously determine the mobile signal(s) for SAR, several components have been identified that contribute to the coordination of pathogen and biotic stress response (Figure 4, right).

Comparative proteomics uncovered 16 proteins induced in phloem exudates after SAR induction. Conversely, the same study also found that 46 phloem proteins are suppressed under SAR [76]. Some of the identified SAR-responsive phloem proteins, like PATHOGENESIS RELATED 1 (PR1) and APOPLASTIC ENHANCED DISEASE SUSCEPTIBILITY 1-DEPENDENT 15 (AED15) are hypothesized to act as antimicrobial or anti-herbivory agents in phloem sap directly. However, other proteins are thought to be important for SAR long-distance signaling, either as signal carriers or as signals themselves [76]. Among these are several predicted lipid-interacting proteins, including DEFECTIVE IN INDUCED RESISTANCE 1 (DIR1), Acyl-CoA Binding Proteins (ACBPs), and a major latex-like protein (MLP). In addition, several lipophilic compounds such as dehydroabietinal (DHA), azelaic acid (AZA), and glycerol-3-phosphate (G3P) derivatives have been found to play a role in SAR [12,129, 130, 133, 135]. The accumulation of these proteins in the phloem during SAR suggests a role for lipid-binding proteins in conveying a lipophilic compound as part of the SAR signal.

DIR1 is a lipid transfer protein that has been shown to be phloem mobile during SAR and is required for systemic resistance [79,183]. While DIR1 alone does not induce resistance in distant tissues, it may be required for the transport of other SAR signals like DHA [131]. Additionally, AZELAIC ACID INDUCED 1 (AZI1) and EARLY ARABIDOPSIS ALUMINUM INDUCED 1 (EARLI1) may play a role in the loading and transport of AZA as a mobile SAR signal [134].

Thioredoxin H (TRXH) proteins have been identified in phloem sap during SAR in *Arabidopsis* as well as other plants species like rice [76,138]. While the function of TRXH in the phloem is unknown, it has been linked to pathogen immunity by regulating the oligomerization of NONEXPRESSER OF PR GENES 1 (NPR1) [184]. It is possible that the phloem-localized TRXH protein could act to regulate other mobile SAR signals, like DIR1.

Among the phloem-localized, wound-response genes are acyl-CoA binding proteins *ACBP3* and *ACBP6. ACBP6* is expressed in CCs in response to wounding, and the protein is found in phloem exudates [136]. The same is true for ACBP3. Grafting studies indicate that ACBP3 is phloem-mobile and moves from shoots to roots. Furthermore, the absence of *ACBP3* impairs defense response in both locally wounded and distal tissue [137]. ACBPs play a role in maintaining acyl-CoA pools and lipid metabolism. Along these same lines, both ACBP3 and ACBP6 affect the fatty acid composition of the phloem, more specifically a group of defense-related fatty acids, the oxylipins such as 12-oxo-phytodienoic acid (OPDA) and methyl jasmonate [137]. These oxylipins have long been suggested as mobile signals for wounding, pathogenesis, and SAR [139, 141–143]. Jasmonate is involved both locally and distally for the transmission and perception of systemic wounding signals [144]. The JA precursor OPDA and its derivatives have been shown to translocate via the phloem from wounded shoots to undamaged root tissue, where these precursors are converted into bioactive JA-Ile for systemic defense response [12,140].

As was seen in the previously discussed long-distance signaling paths, a combination of compounds, in this case proteins, small molecules and lipids, are essential to mediate a response. Their exact interplay remains to be determined. In addition, beyond chemical signals, electrical signaling appears to contribute to systemic wound signaling as well. A glutamate/glutamate receptor/calcium ion module acts to propagate signals from local to distal leaves. In this mechanism, Ca^2+^ ions participate as a phloem mobile signal to coordinate wound response systemically [145].

## Water stress and ABA signaling

Systemic signaling for abiotic stress is understudied compared with biotic stress responses like SAR. Nonetheless, hormones, proteins, and peptides in the phloem seem to play an important role in the coordination of systemic abiotic stress response as well (Figure 4, left). One crucial environmental factor/stress is the availability of water. We can distinguish between different water stresses: too much, too little, or inaccessibility due to high salt/osmotic properties of the soil or freezing temperatures. Whether water stress perception predominates in the roots or the leaves is an active area of research [185].

ABA is a phytohormone produced in response to drought-, salt-, osmotic-, and freezing-based water limitation as well as for seed and root development, acting both locally and systemically [186]. The long-distance transport of ABA has been attributed to both the xylem [187–190] and the phloem [146–149], with reports of both root- and shoot-derived ABA pools during water stress [150–152]. One of the water-stress responses, the ABA-mediated stomatal closure is regulated, in part, by a root-derived, vascular-mobile CLAVATA3/EMBRYO-SURROUNDING REGION-RELATED (CLE) protein CLE25. When dehydration is sensed in the roots, *CLE25* is expressed in root vascular tissue, and the protein is transported to leaves where it is sensed by BARELY ANY MERISTEM 1 (BAM1) and 3 receptor-like kinases and affects the expression of an ABA synthesis enzyme *NINE-CIS-EPOXYCAROTENOID DIOXYGENASE 3* (*NCED3*) [153]. Whether the root-to-shoot translocation of CLE25 occurs in xylem or phloem remains to be determined [191] The increase in ABA is sensed by its receptor PYRABACTIN RESISTANCE (PYR)/PYR-LIKE (PYL)/REGULATORY COMPONENTS OF ABA RECEPTOR (RCAR), and the protein phosphatase 2C enzyme ABSCISIC ACID INSENSITIVE 1 (ABI1) is sequestered, in part, by PHOSPHOLIPASE Dα1-derived Phosphatidic acid (PtdOH) [192]. With ABI1 no longer available to dephosphorylate and repress SNF1-RELATED KINASE 2 (SnRK2) activity, SnRK2s can then phosphorylate ABA-Response Element (ABRE) Binding/ABRE Binding Factors (AREB/ABF) TFs, which initiate the expression of drought-responsive genes by interacting with ABA response elements in promoter regions. These findings indicate that both, the hormone ABA as well as the protein CLE25 are long-distance signals mediating the water-stress response. Alternatively, hydraulic signals have been proposed instead of root-derived ABA as an indicator of water limitation [193].

Recent data suggest that protein–lipid complexes may be involved in drought signaling as well. Several small lipid-binding proteins have been identified in the phloem and were proposed to play a role in long-distance signaling [12,132,154]. PHLOEM LIPID ASSOCIATED FAMILY PROTEIN (PLAFP) may be involved in drought response and ABA signaling. *PLAFP* is expressed in the vasculature. Its expression increases in response to treatment with ABA as well as the drought mimic polyethylene glycol [154]. PLAFP interacts specifically with PtdOH, a known stress signal involved in ABA signaling and detected in phloem exudate [12,192,154, 194–196]. These data suggest the PLAFP–PtdOH complex as a possible phloem-mobile signal for the systemic coordination of drought response [132,155].

These findings further substantiate previous results that long distance signaling, rather than relying on single molecules, often encompasses multiple converging pathways and the interaction between proteins, RNA, small molecules, and even lipids.

## Conclusion

The last few decades have shown that the plant phloem is far more complex than originally anticipated: in addition to sugars, it contains a vast array of small molecules, hormones, proteins, lipids, and nucleic acids that facilitate tightly coordinated developmental and stress responses. The heterogeneity of systemic-driven processes requires a diversity of phloem-associated molecular species. The examples discussed in this review aim to highlight this multiplicity: it is not a single molecule or class of molecules that predominates long-distance signaling, but rather a diverse arsenal of signal classes interact to systemically coordinate plant development. The role of these, often multipronged, signaling processes remains to be resolved—perhaps they confer redundancy to ensure some minimum response or modularity to fine-tune the intensity and specificity of the response. While much has been learned, phloem signaling continues to be an important field of active research essential not only to understand how plants respond to changes in the environment but also to ensure food security.

## Abbreviations

AAP: amino acid permease
ABA: abscisic acid
ABF: ABA-response element binding factor
ABI1: abscisic acid insensitive 1
ABRE: ABA-response element
ACBP: Acyl-CoA binding protein
AED15: apoplastic enhanced disease susceptibility 1-dependent 15
AREB: ABA-Response Element Binding
ATC: *arabidopsis* centroradialis
AZA: azelaic acid
AZI1: aza induced 1
BAM1: barely any meristem 1
BEL: bel 1-related homeotic proteins
CC: companion cell
CEP: c-terminally encoded peptide
CEPD: cep downstream
CEPDL2: cepd-like 2
CEPR: cep receptor
CK: cytokinin
CLE25: clavata3/emryo-surrounding region-related
CO: constans
CYP1: cyclophilin 1
DHA: dehydroabietinal
DIR1: defective in induced resistance 1
FAC: florigen activation complex
FRO2: ferric reduction oxidase 2
FT: flowering locus T
FTIP1: FT interacting protein 1
G3P: glycerol-3-phosphate
GA: gibberellin
HY5: elongated hypocotyl 5
IAA: indole-3-acetic acid
IPS1: induced by phosphate starvation 1
IRT1: iron-regulated transporter 1
JA: jasmonic acid
MCTP-SNARE: multiple C2 domain and transmembrane protein-soluble *N*-ethylmaleimide-sensitive factor protein attachment protein receptor
miR2111: microRNA 2111
miR399: microRNA 399
miRNA: microRNA
MLP: major latex-like protein
NCED3: nine-cis-epoxycarotenoid dioxygenase 3
ncRNA: non-coding RNA
NPR1: nonexpressor of pr genes 1
NRT2.1: nitrate transporter 2.1
OPDA: 12-oxo-phytodienoic acid
OPT3: oligopeptide transporter 3
PD: plasmodesmata
PHO2: phosphate 2
PLAFP: phloem lipid-associated family protein
POTH1: potato homeobox 1
PR1: pathogenesis related 1
PtdCho: phosphatidylcholine
PtdOH: phosphatidic acid
PYR/PYL/RCAR: pyrabactin resistance/pyr-like/regulatory components of aba receptor
QKY: quirky
SAM: shoot apical meristem
SAR: systemic acquired resistance
SE: sieve element
SFT: single flower truss
siRNA: small interfering RNA
SnRK2: snif1-related kinase 2
StSP6A: *solanum tuberosum* self-pruning 6A
SUT: sucrose transporter
SWEET: sugar will eventually be exported transporter
SYP121: syntaxin of plants 121
TF: transcription factor
TML: too much love
TRXH: thioredoxin H
UMAMIT: usually multiple acids move in and out transporter
